# Immobilization of *Moniliella spathulata* R25L270 Lipase on Ionic, Hydrophobic and Covalent Supports: Functional Properties and Hydrolysis of Sardine Oil

**DOI:** 10.3390/molecules22101508

**Published:** 2017-09-25

**Authors:** Lívia T. de A. Souza, Sonia Moreno-Perez, Gloria Fernández Lorente, Eliane P. Cipolatti, Débora de Oliveira, Rodrigo R. Resende, Benevides C. Pessela

**Affiliations:** 1 Departamento de Bioquímica e Imunologia, Universidade Federal de Minas Gerais, Avenida Presidente Antônio Carlos, 6627, Caixa Postal 486, Belo Horizonte MG 31270-901, Brazil; 2 Pharmacy and Biotechnology Department, School of Biomedical Sciences, Universidad Europea, Villaviciosa de Odón, 28670 Madrid, Spain; chaspy_27@hotmail.com; 3 Departamento de Biotecnología y Microbiología de Alimentos, Instituto de Investigación en Ciencias de la Alimentación CIAL (CSIC-UAM), Campus de la Universidad Autónoma de Madrid, Nicolás Cabrera 9, 28049 Madrid, Spain; gflorente@csic.es; 4 Departamento de Engenharia Química e Engenharia de Alimentos, Universidade Federal de Santa Catarina (UFSC), P.O. Box 476, Florianópolis SC 88040-900, Brazil; elianecipolatti@yahoo.com.br (E.P.C.); debora.oliveira@ufsc.br (D.d.O.); 5 Instituto Nanocell, Divinópolis MG 35500-041, Brazil; 6 Departamento de Engenharia e Tecnologías, Instituto Superior Politécnico de Tecnologías e Ciências (ISPTEC) Av. Luanda Sul, Rua Lateral Via S10, P.O. Box 1316, Talatona-Luanda Sul, Angola

**Keywords:** lipase, *Moniliella spathulata*, immobilization, fish oil, omega-3

## Abstract

The oleaginous yeast *Moniliella spathulata* R25L270 was the first yeast able to grow and produce extracellular lipase using Macaúba (*Acrocomia aculeate*) cake as substrate. The novel lipase was recently identified, and presented promising features for biotechnological applications. The *M. spathulata* R25L270 lipase efficiently hydrolyzed vegetable and animal oils, and showed selectivity for generating *cis*-5,8,11,15,17-eicosapentaenoic acid from sardine oil. The enzyme can act in a wide range of temperatures (25–48 °C) and pH (6.5–8.4). The present study deals with the immobilization of *M. spathulata* R25L270 lipase on hydrophobic, covalent and ionic supports to select the most active biocatalyst capable to obtain omega-3 fatty acids (PUFA) from sardine oil. Nine immobilized agarose derivatives were prepared and biochemically characterized for thermostability, pH stability and catalytic properties (K_M_ and V_max_). Ionic supports improved the enzyme–substrate affinity; however, it was not an effective strategy to increase the *M. spathulata* R25L270 lipase stability against pH and temperature. Covalent support resulted in a biocatalyst with decreased activity, but high thermostability. The enzyme was most stabilized when immobilized on hydrophobic supports, especially Octyl-Sepharose. Compared with the free enzyme, the half-life of the Octyl-Sepharose derivative at 60 °C increased 10-fold, and lipase stability under acidic conditions was achieved. The Octyl-Sepharose derivative was selected to obtain omega-3 fatty acids from sardine oil, and the maximal enzyme selectivity was achieved at pH 5.0.

## 1. Introduction

The oleaginous yeast *Moniliella spathulata* R25L270, isolated from the Brazilian butter cheese (Requeijão do Norte) (Minas Gerais, Brazil), was the first yeast able to be grown and produce extracellular lipase using Macaúba (*Acrocomia aculeate*) cake as substrate [1]. The lipase from *M. spathulata* R25L270 exhibited at least 80% of the maximal activity value by using different combinations of temperature (25–48 °C) and pH (6.5–8.4) ranges [1]. Moreover, the lipase efficiently hydrolyzed various vegetable oils, and showed selectivity for generating *cis*-5,8,11,15,17-eicosapentaenoic acid (EPA) from sardine oil [1]. The enzyme was purified and the first lipase from *M. spathulata* R25L270 was identified by mass spectrometry [1]. Research concerning lipase production by new microbial sources was stimulated decades ago, mainly because of the industrial importance of lipases, especially in the food and biodiesel sectors [2,3,4,5]. Lipase (triacylglycerol acyl hydrolases EC 3.1.1.3) refers to a class of enzymes which can catalyze the hydrolysis of triacylglycerol to free fatty acids and glycerol [6,7]. They are usually used in an organic medium catalyzing esterification, transesterification and interesterification reactions [6,7]. An important application of lipases is to obtain polyunsaturated fatty acids (PUFA) via enzymatic hydrolysis of fish oil, which is rich in *cis*-5,8,11,15,17-eicosapentaenoic acid (EPA) and *cis*-4,7,10,13,16,19-docosahexaenoic acid (DHA) [8,9,10,11,12,13]. The use of enzymes may be favorable compared to chemical processes by being more specific, decreasing steps in production and reducing the generation of undesirable compounds [8,9,10,11,12]. Omega-3 fatty acids are physiologically essential nutrients required in all stages of life, playing a significant role in human health. For example, in children, the brain and the retina require high levels of DHA, which improves learning ability, mental development and visual acuity [14,15]. In adults, EPA is considered to be useful in the prevention of cardiovascular diseases [14,15]. Unfortunately, difficulty in the separation and recovery of free lipase from solution after the hydrolysis process limits the reusability of the enzyme, which makes impracticable the scale-up of this application because of the high cost of the biocatalyst [5,16]. Enzyme immobilization is one effective way to allow enzymes to be reused, and therefore, to reduce the cost of the bioprocess [16,17]. Researchers have applied many kinds of materials as carriers for the immobilization of lipases by different immobilization methods, such as epoxy–polysiloxane–polyvinyl alcohol composite) [18]; polyurethane with polyethylene glycol [19]; hydrophobic magnetic microparticles [20]; agarose supports [8,10]; styrene–divinylbenzene beads [21]; chitosan [22] and aluminum oxide pellets [23]. The selection of immobilized carriers plays an important role in enzyme immobilization, since it significantly influences the properties of the immobilized biocatalysts. The objective of the present work was to immobilize *M. spathulata* R25L270 lipase on hydrophobic, covalent and ionic supports to be used for PUFA production by fish oil hydrolysis.

## 2. Results and Discussion

### 2.1. Immobilization of M. spathulata R25L270 Lipaseon Different Supports

*M. spathulata* R25L270 was grown using Macaúba cake as substrate, and the culture supernatant—containing 0.6 ± 0.1 U/mL of lipolytic activity—was used as a source of free lipase in the immobilization assays using hydrophobic, covalent and ionic supports. The immobilization methods and immobilized carrier significantly influenced the immobilization kinetic parameters, as shown in Table 1.

When *M. spathulata* R25L270 lipase was immobilized by adsorption on hydrophobic supports, the immobilization yields (Y) for all prepared biocatalysts were around 60%, and the maximum recovered activity (95.8%) was achieved using Butyl-Sepharose. These results suggest that lower hydrophobicity of the carrier promoted less distortion on the three-dimensional structure of lipase, or better orientation on the support surface that facilitates the access of *p*-nitrophenylpalmitate (pNPP) to their active sites. *Hypocrea pseudokoningii* lipase exhibited the same behavior when immobilized on hydrophobic supports [24]. The enzyme was even activated when immobilized on Butyl-Sepharose (the relative activity of the prepared biocatalyst to the free lipase was 1.55), however, the lipase presented lower activity on decaoctyl-Sephabeads, the most hydrophobic support evaluated [24]. The subsequent treatment of the Octyl-Sepharose derivative with stabilizing agents trehalose or polyethyleneimine (PEI) did not show prominent effects on kinetic parameters of immobilization. Hydrophobic adsorption at low ionic strength is perhaps the simplest method of lipase immobilization, purification and stabilization [1,25,26,27,28]. Moreover, studies carried out in our lab concerning the immobilization of different commercial lipase formulations often led to enhancement of the catalytic activity of the lipase [28,29]. This is interpreted as being due to hydrophobic interactions between the support and the hydrophobic surroundings of the enzyme active site, causing opening of the lid to yield the active conformation, analogous to the interfacial activation caused by a natural substrate [28,29].

The immobilization of *M. spathulata* R25L270 lipase by adsorption on ionic supports was studied using anion- and cation-exchanger carriers. Both MANAE (monoaminoethyl-*N*-aminoethyl)-agarose and DEAE (diethylaminoethyl)-Sepharose are anion-exchanger resins, with amino groups added to agarose. In this way, they are capable of immobilizing proteins via their carboxyl terminal group or via negatively charged residues at a particular pH. On the other hand, SF (sulfopropyl)-Sepharose allows immobilization via protein amino terminal groups or positively charged residues at a given pH. As shown in Table 1, *M. spathulata* R25L270 lipase immobilization was possible only with anion-exchanger supports, with immobilization yields similar to hydrophobic supports (around 70%). However, low activity could be recovered in the derivatives, especially MANAE-agarose, which may be related to enzyme distortion during immobilization. The negatively charged groups of this enzyme at pH 7.0 demonstrated importance for the maintenance of protein structure, and their involvement in immobilization promoted a strong distortion on the three-dimensional structure of the lipase or bad orientation on the support surface that restricted access of the substrate to their active sites. *H. Pseudokoningii* lipase also showed high density of negatively charged groups on the enzyme surface, and could be immobilized on Q-Sepharose, PEI-agarose, DEAE-agarose and MANAE-agarose, with better immobilization performance obtained for Q-Sepharose [24].

*M. spathulata* R25L270 lipase allowed for a low immobilization yield (36.27%) and recovered activity (22.73%) when irreversibly immobilized on a CNBr (Cyanogenogen Bromide)-activated Sepharose support. At immobilization conditions of pH 7.0, at 25 °C and for 24 h, it is expected that a multipoint covalent attachment is established between the terminal amine group, which has a p*K*_a_ around 7–8, and reactive amino groups positioned on the enzyme surface. Although lower activity has been noted, the covalent immobilization provides biocatalysts with high rigidity [16,30].

The immobilization kinetic parameters found for *M. spathulata* R25L270 lipase demonstrated that physical adsorption, especially hydrophobic adsorption, has the advantage of high retention of enzymatic activity.

### 2.2. Characterization of M. spathulata R25L270 Lipase Immobilized Derivatives

#### 2.2.1. Apparent Kinetic Parameters (V_max_ and K_M_)

The influence of immobilization on the catalytic properties of *M. spathulata* R25L270 lipase was studied by comparing the values of the apparent V_max_ and K_M_ for free and immobilized lipase using pNPP as substrate. The results demonstrated that free and immobilized *M. spathulata* R25L270 lipase obeyed Michaelis–Menten kinetics (*R*^2^ ≥ 0.97) (Table 2). The interaction of *M. spathulata* R25L270 lipase with the support had a relevant effect on the accessibility of the substrate to the active center of the enzyme, resulting in changes in the recognition of the lipase by pNPP. As shown in Table 2, the values of apparent K_M_ found for the CNBr-activated Sepharose derivative and the DEAE-Sepharose derivative were 0.10 mM and 0.40 mM, respectively, while for free lipase it was 1.92 mM. These results demonstrated that the affinity of the *M. spathulata* R25L270 lipase for the pNPP substrate can be increased by the type of carrier used in the immobilization step. The immobilization of *M. spathulata* R25L270 lipase in the Octyl-Sepharose hydrophobic support did not result in prominent changes in the affinity of the enzyme for the substrate, even when the support was treated with the stabilizing agents trehalose and PEI after the immobilization step. The apparent V_max_ value for the free lipase was 3.15 µmoles/min, which decreased after immobilization—especially for the CNBr-activated Sepharose—to 1.03 µmoles/min. This data set demonstrated that the immobilization of the same enzyme in different immobilization supports results in biocatalysts with completely different catalytic properties. This was also observed for *Candida rugosa* lipase. Dandavate et al., using pNPP as substrate, found *C. rugosa* lipase K_M_ values of 1.43 mM and 0.3 mM for free and lipase-immobilized on nanoparticles, respectively [31]. Živković et al. reported similar K_M_ values for *C. rugosa* lipase (free or immobilized in zirconia), demonstrating no change in enzyme–substrate affinity [32]. On the other hand, when *C. rugosa* lipase was immobilized in membranes, an approximately 7-fold decrease in enzyme–substrate affinity was found [33]. An even larger decrease in V_max_ (21 times) was reported for *C. rugosa* lipase immobilized in niobium oxide [34]. The value of V_max_ for *C. Rugosa* lipase immobilized in silica and zirconia was 4.8-fold and 3.6-fold lower, respectively, than that of the free lipase [32]. Reduced activity of the immobilized enzymes has been considered to be a consequence of mass transfer limitations and the rigidity of immobilized proteins [32,34].

#### 2.2.2. pH Stability

*M. spathulata* R25L270 lipase is versatile, and has potential for applications in bioprocesses that occur in pH range 6.5–8.4 [1]. The free enzyme remained stable in neutral and alkaline conditions, with loss of approximately 40% of its initial activity after incubation with citrate buffer (pH 5.0) at 25 °C for 4 h (Table 3). The immobilization of *M. spathulata* R25L270 lipase by hydrophobic adsorption on Phenyl-Sepharose and Octyl-Sepharose supports increased enzyme stability in acidic conditions, and the residual enzymatic activity was higher than 80%. On the other hand, the immobilization of *M. spathulata* R25L270 lipase in Butyl-Sepharose, CNBr-activated Sepharose and DEAE-agarose supports resulted in total loss of enzyme activity at pH 5.0. Under neutral and alkaline conditions, the enzyme could be activated by immobilization, as found for the CNBr-activated Sepharose derivative (Table 3). The immobilization of *M. spathulata* R25L270 lipase by ionic adsorption on the DEAE-agarose support resulted in a biocatalyst with differentiated properties. As shown in Table 3, the biocatalyst was more stable at pH 9.0, probably due to the most-favourable interaction established between the negatively-charged groups of the enzyme surface and the positively-charged groups on the support. The pH stability of *M. spathulata* R25L270 lipase could be altered by the immobilization technique. The same behaviour was found for *C. rugosa* lipase.The free enzyme remained stable in the pH range from 3 to 6, while the immobilized lipases were found to be stable up to a pH of 7.0 [33]. The effect of pH on enzyme stability could be explained by the interference in the net electric charge of the protein or support, modifying the electrostatic forces that stabilize protein structure and/or the protein–support interaction [16,17].

#### 2.2.3. Thermostability

*M. spathulata* R25L270 lipase has potential for applications in bioprocesses that occur in the temperature range 25–48 °C [1]. It is well known that immobilization techniques can increase enzyme thermostability, stimulating the industrial application of the biocatalyst. The thermostability of *M. spathulata* R25L270 lipase was studied at 60 °C at pH 7.0, and the deactivation rate constant (K_d_) and the half-life time (*t*_1/2_) were determined by applying the exponential non-linear decay model proposed by Sadana and Henley [35].

**Figure 1:**
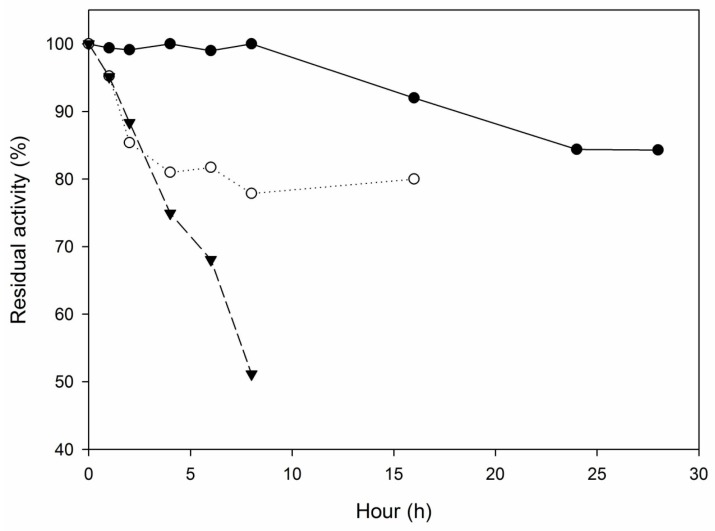
Octyl + PEI (▼), Octyl+trehalose (○) and CNBr-activated Sepharose (●) derivatives thermal stability at 60 °C. Incubation was carried out in 5 mM sodium phosphate buffer, pH 7.0, under non-reactive conditions.

The half-life found for *M. spathulata* R25L270 free lipase at 60 °C was 0.40 h. In general, the immobilization of lipase resulted in more thermostable biocatalysts. As shown in Table 4, with the exception of the DEAE-agarose derivative (stabilization factor (SF) < 1), all immobilized derivates exhibited superior residual activity (SF > 1) compared to the free lipase. For *M. spathulata* R25L270 lipase immobilized by hydrophobic adsorption, when support hydrophobicity was increased, the enzyme exhibited significant resistance to the thermal inactivation. The Octyl-Sepharose derivative was around 10-fold more stable than the free lipase, probably due to the strong interaction between enzyme and support, culminating in less flexibility of the protein. The stabilizing agent trehalose had a prominent effect on *M. spathulata* R25L270 lipase stabilization, without severe loss of enzyme activity, even after long incubation time (Figure 1). The stabilizing agent PEI also had an effect on enzyme thermostability. The thermal decay profile of theoctyl + PEI derivative differed from the others, and therefore, the kinetic parameters could not be determined using the model proposed. At 6 h of incubation, the residual activities found for Octyl-Sepharose, octyl + PEI and octyl + trehalose derivatives were 53.26%, 51.14% and 82.53%, respectively; thus, immobilization by adsorption on hydrophobic supports was showed to be a successful alternative for *M. spathulata* R25L270 lipase stabilization.

The covalent immobilization of *M. spathulata* R25L270 lipase on the CNBr-activated Sepharose support resulted in a biocatalyst with greater stability at 60 °C (Figure 1). The derivative retained almost 80% of the initial activity, even after 30 h of incubation at 60 °C, revealing itself as one of the most powerful strategies to thermally stabilize *M. spathulata* R25L270 lipase. It is known that covalent immobilization may produce a strong rigidification of the enzyme structure, mainly if a very intense multipoint covalent attachment is achieved [16,36].

Compared to other fungal lipases, *M. spathulata* R25L270 lipase, even in soluble form, demonstrated great thermostability. The biocatalysts prepared by immobilization of *Hypocrea pseudokoningii* lipase on hydrophobic supports were strongly inactivated when incubated at 60 °C, except for the immobilized lipase on Octyl-Sepharose, which showed a half-life of 110 min [24]. *C. rugosa* lipase immobilized in membranes exhibited superior activity in contrast to the free lipase when incubated at 60 °C for 60 min. The immobilized enzyme remained with 90.5% activity, whereas the activity of free lipase was just 8% [33]. When *C. rugosa* was immobilized on dry and wet chitosan beads, the activities found after 60 min at 60 °C were 45% and 60%, respectively; whereas the activity retained by the free enzyme was only 20% [22].

### 2.3. Hydrolysis of Sardine Oil by M. spathulata R25L270 Lipase Immobilized on Octyl-Sepharose

The potential application of *M. spathulata* R25L270 free lipase to catalyse vegetable and sardine oils was recently published [1]. The enzyme preferentially catalysed sesame, olive and sunflower oils, and exhibited interesting catalytic properties with regard to selective hydrolysis of sardine oil [1]. It is well known that the immobilization technique may alter the selectivity of the enzymes. Due to high lipase activity and improved stability, Octyl-Sepharose derivatives were selected to catalyze the release of EPA (eicosapentaenoic acid) and DHA (docosahexaenoic acid) omega-3 fatty acids from the hydrolysis of sardine oil. The effect of stabilizing agents trehalose and PEI also was studied. A biphasic system of water–cyclohexane was used in order to make oil manipulation feasible. In this case, lipase inside the pores of supports could only hydrolyze the oil molecules partitioned in the aqueous phase of the system, as proposed by Fernández-Lorente et al. [11].

**Figure 2:**
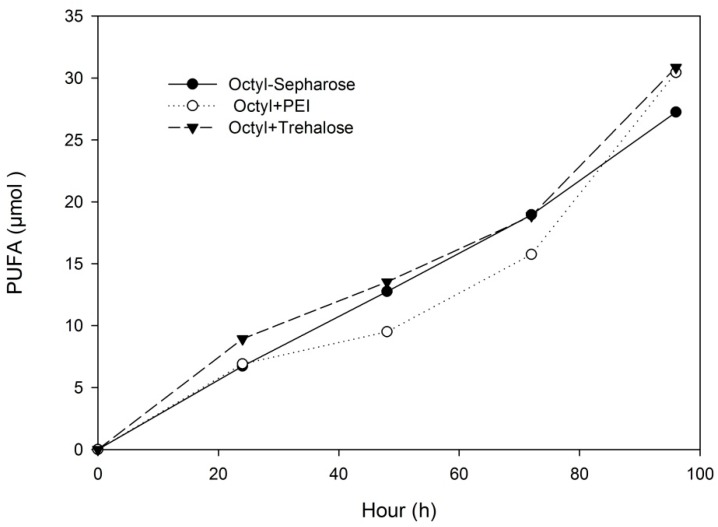
Release rates of PUFA (EPA + DHA). Reaction was carried out in an aqueous/organic biphasic system, with 50 mM sodium phosphate buffer pH 7.0/cyclohexane, at 37 °C and 150 rpm. EPA: eicosapentaenoic acid; PUFA: polyunsaturated fatty acids; DHA: docosahexaenoic acid.

The release rates of polyunsaturated fatty acids (PUFA) were quite similar for all biocatalysts assayed, and the treatment of the Octyl-Sepharose derivative with trehalose or PEI was not very effective in improving reaction yields and selectivity when the reaction was conducted in 50 mM sodium phosphate buffer pH 7.0 (Figure 2 and Table 5). With the same reaction conditions, the immobilization of lipase resulted in a decrease of selectivity compared to the free enzyme, which was estimated to be 4.97 [1]. The hydrolysis reaction using immobilized lipase was also conducted at pH 5.0 and pH 9.0. The pH of the reaction medium was shown to be a variable of great influence on enzyme selectivity. Under acidic conditions, an increase in enzyme selectivity was achieved, especially for Octyl-Sepharose (Table 5). At pH 5.0, the production of 7.53:1 EPA:DHA mixtures from sardine oil hydrolysis was obtained.

The performance of the lipase under study against the hydrolysis of sardine oil was comparable to commercially available lipases. The selectivity found for commercial lipases *Rhizomucor miehei* lipase (RML), *Candida antarctica* lipase fraction B (CALB) and *Thermomyces lanuginosa* lipase (TLL), adsorbed on Octyl-Sepharose and acting in a biphasic reactive medium (pH 7.0) containing sardine oil, was 4.47, 1.5 and 4.45, respectively [11]. The application of lipases in the food industry to obtain PUFA is not recent, but the possibility of using *M. spathulata* R25L270 immobilized lipases is being investigated for the first time. The potential use of *M. spathulata* R25L270 free and immobilized lipase to be applied in the food industry demonstrates the importance of finding new biocatalysts.

## 3. Materials and Methods

### 3.1. Materials

*M. spathulata* R25L270 strain was obtained from the culture collection of the Universidade Federal de Minas Gerais (Belo Horizonte, Brazil). Macaúba cake was obtained from Biodiesel Company (Minas Gerais, Brazil). The fish oil was obtained from BTSA, Biotecnologías Aplicadas, S.L. (Madrid, Spain), DEAE-agarose (Diethylaminoethyl-agarose), Octyl-Sepharose™ CL-4B, Phenyl-Sepharose™ CL-4B, Butyl-Sepharose, Sulfopropyl-Sepharose and Cyanogenogen Bromide (BrRCN)(CNBr) supports were obtained from GE Bio-Sciences AB (Uppsala, Sweden). The substrate pNPP and all other chemicals were obtained from Sigma Chemical Co. (St. Louis, MO, USA).

### 3.2. Lipase Production using Macaúba Cake as Substrate

*M. spathulata* R25L270 is a yeast strain isolated from Brazilian butter cheese (Requeijão do Norte, Minas Gerais, Brazil) [1]. The yeast was cultivated on Sabouraud agar slants at 30 °C for 96 h. For inoculum preparation, fresh cells were transferred to 125 mL Erlenmeyer flasks containing 25 mL medium composed of glucose (20.0 g/L), peptone (10.0 g/L) and yeast extract (5.0 g/L). The inoculated flasks were incubated at 30 °C at 200 rpm for 24 h, after which the cells were recovered by centrifugation (5000× *g*, 10 min) at room temperature (25 ± 1 °C) and suspended in the fermentation medium at the desired initial culture density (DO600 = 0.1). For lipase production, the fresh cells were inoculated into 500 mL Erlenmeyer flasks containing 100 mL of the culture medium NH_4_NO_3_ (1.0 g/L); KH_2_PO_4_ (1.0 g/L); MgSO_4_·7H_2_O (5.0 g/L); peptone A (20.0 g/L) and Macaúba cake (40.0 g/L). The fermentation was carried at 30 °C, pH 6.5 and continuous agitation of 200 rpm. After 120 h of cultivation, cultures were centrifuged (8000× *g*, 15 min) at room temperature (25 ± 1 °C) and the resulting supernatant was used as source of crude free lipase. The supernatant was stored under refrigeration (−20 °C) for late use within 1 month.

### 3.3. Lipase Activity Assay

Enzyme activity was measured by monitoring spectrophotometrically the increase in absorbance at 410 nm, produced by the release of *p*-nitrophenol (pNP) in the hydrolysis of *p*-nitrophenylpalmitate (pNPP). For the hydrolytic assay, the substrate solution was prepared by mixing 1 mL of solution A (90 mg of pNPP dissolved in 30 mL 2-propanol) and 9 mL of solution B (90 mM Tris-HCl buffer (pH 8.0); 2.0% Triton X-100; and 0.2% gum arabic) [37]. All analyses were performed in triplicate. One unit of lipase (U) was defined as the amount of soluble enzyme (mL) or prepared biocatalyst (g) that releases 1 µmol *p*-nitrophenol (pNP) per min in the assay conditions (pH 8.0, 37 °C). The lipolytic activity of each derivative corresponded to the measurement of the derivative enzyme activity per gram of support (U/g).

### 3.4. Immobilization of M. spathulata R25L270 Lipase by Adsorption on Hydrophobic and Ionic Supports

The culture supernatant containing 0.6 ± 0.1 U/mL of lipolytic activity was used as a source of free lipase in immobilization assays. Three hydrophobic supports (Butyl-, Phenyl- and Octyl-Sepharose) and three ionic supports (monoaminoethyl-*N*-aminoethyl (MANAE)-agarose, diethylaminoethyl (DEAE)–Sepharose and sulfopropyl (SP)-Sepharose) were evaluated to immobilize *M. spathulata* R25L270 lipase. The immobilization was performed by the mixture of 8.0 mL of culture supernatant, 2.0 mL of 5 mM phosphate buffer pH 7.0 and 1.0 g of support. Lipase support systems were kept in contact for 4 h at 25 °C under mild stirring, and then the biocatalysts were vacuum filtrated with the aid of a sintered funnel and washed with 5 mM phosphate buffer pH 7.0. All biocatalysts prepared were stored at 4 °C. Trehalose and polyethyleneimine (PEI) were studied as potential stabilizing agents after the immobilization step using the Octyl-Sepharose support. For this, the biocatalyst Octyl-Sepharose was incubated for 2 h at 25 °C under mild stirring with 50 mM sodium phosphate buffer pH 8.0 containing trehalose or polyethyleneimine (PEI) at a concentration of 1% *p*/*v*.

### 3.5. Immobilization of M. spathulata R25l270Lipase by Covalent Attachment on CNBr-Activated Sepharose

The CNBr-activated Sepharose support was prepared following the manufacturer’s instructions. Dry resin was swelled and activatedin 35 mL of 0.1 M HCl, pH 2.0 for 45 min. Then, the swollen support was washed with the same acid solution and vacuum filtrated with the aid of a sintered funnel. The immobilization was performed by the mixture of 8.0 mL of culture supernatant, 2.0 mL of 5 mM phosphate buffer pH 7.0 and 1.0 g of support. Lipase support systems were kept in contact for 4 h at 25 °C under mild stirring, and then the biocatalysts were vacuum filtrated with the aid of a sintered funnel and washed with 5 mM phosphate buffer pH 7.0. The biocatalysts prepared were stored at 4 °C.

### 3.6. Determination of Immobilization Kinetic Parameters

The immobilization kinetic parameters were calculated based on the measurement of enzyme activity before and after immobilization steps. The total lipase activity offered for each support was approximately 5 U. The immobilization yields (*Y*) and recovered activity (*RA*) were calculated using Equations (1) and (2), respectively.

(1) $$\mathrm{Immobilization}\;\mathrm{yield}\;(\%)\;(Y)=\frac{A-B}{A}\times 100$$

where *A* is the total of lipase activity offered for each support and *B* is the total of lipase activity found in the supernatant at the end of immobilization step.

(2) $$\mathrm{Recovered}\;\mathrm{activity}\;(\%)\;(RA)=\frac{C}{A\times Y}\times 100$$

where *C* is the total of lipase activity found in the support, *A* is the total of lipase activity offered for each support and *Y* is the immobilization yield for that support.

### 3.7. Biochemical Characterization of M. Spathulata R25L270 Lipase Immobilized on Different Supports

#### 3.7.1. Kinetic Parameters Estimation

The influence of immobilization on the catalytic properties of *M. spathulata* R25L270 lipase was analyzed in pNPP solutions at concentrations varying from 0.1 to 1 mM at 37 °C and pH 8.0. For this, a lipase suspension was prepared by suspending 0.1 g of each immobilized derivative in 1 mL of 50 mM sodium phosphate buffer pH 7.0. Values for apparent K_M_ and V_max_ were calculated based on the Michaelis–Menten equation using the computational program Sigma Plot software 10.0. Experiments were performed in triplicate, and crude free lipase was used as a control.

#### 3.7.2. pH Stability

Prepared biocatalysts (0.1 g) were incubatedin 1 mL of 50 mM McIlvaine buffer for pH 5.0; 50 mM sodium phosphate buffer pH 7.0; and 50 mM bicarbonate buffer for pH 9.0. Experiments were performed at 25 °C in triplicate, and the hydrolytic activities were determined before and after 4 h of incubation. The results were expressed as a percentage of initial activity.

#### 3.7.3. Thermostability

Prepared biocatalysts (0.1 g) were incubated in 1 mL of 50 mM sodium phosphate buffer pH 7.0 at 60 °C. Samples of lipase suspension were periodically withdrawn and their residual activity was measured using the pNPP assay. The residual activity was calculated as the ratio between the activity at a given time and the activity at time zero of incubation. The observed deactivation rate constant (K_d_) and the half-life time (*t*_1/2_) of each prepared biocatalyst were determined by applying the exponential non-linear decay model proposed by Sadana and Henley [35]. The experimental data were analyzed using Sigma Plot software, version 10.0. Stabilization factors (SF) were obtained as the ratio between the half-lives of the prepared biocatalysts and the crude free lipase supernatant, as shown in Equation (3).

(3) $$\mathrm{Stabilization}\;\mathrm{factors}\;(\mathrm{SF})=\frac{t_{1/2}^{immob}}{t_{1/2}^{free}}.$$

### 3.8. Sardine Oil Hydrolysis in Biphasic System

Sardine oil hydrolysis was performed with Octyl-Sepharose, Octyl + trehalose and Octyl + PEI biocatalysts prepared. The fish oil used was composed of triacylglycerol formed by 18% eicosapentaenoic acid (EPA) and 12% docosahexaenoic acid (DHA). The reactions were carried out in a glass reactor containing 5.0 mL of substrate consisting of 2.25 mL of cyclohexane, 2.5 mL of buffer and 0.25 mL of sardine oil. Three pH conditions were evaluated: 50 Mm McIlvaine buffer for pH 5.0; 50 mM sodium phosphate buffer pH 7.0; and 50 mM bicarbonate buffer for pH 9.0. The mixtures were incubated with the prepared biocatalysts at a fixed proportion 1 IU per 5.0 mL of substrate under agitation (150 rpm) at 37 °C. The reaction progress was monitored by taking samples at various time intervals, and EPA and DHA released were analyzed using HPLC (Spectra Physic SP 100 coupled with a UV detector Spectra Physic SP 8450) using a reversed-phase column (Ultrabase C18, 4.6 mm i.d. × 150 mm, 5 μm particle size). The concentration of free fatty acids in the organic phase was determined by RP–HPLC (Spectra Physic SP 100 coupled with a UV detector, Spectra Physic SP 8450) using a reversed-phase column (Ultrabase C18, 4.6 mm i.d. × 150 mm, 5 μm particle size). Products were eluted at a flow rate of 1.0 mL/min with acetonitrile:water:acetic acid (70:30:0.1 *v*/*v*/*v*) pH 3. The UV detection was performed at 215 nm, following previous established conditions [11]. All analyses were performed in duplicate.

## 4. Conclusions

*M. spathulata* R25L270 lipase could be immobilized on ionic, hydrophobic and covalent supports, resulting in biocatalysts with different functional properties. Adsorption on hydrophobic supports resulted in high-activity biocatalysts, and the hydrophobic derivatives were more stable to temperature than the free lipase. For example, *t*_1/2_ for Octyl-Sepharose was 4.15 h against 0.40 for free enzyme at pH 7.0. Moreover, the stabilizing agent trehalose had a prominent effect on *M. spathulata* R25L270 lipase stabilization, not showing loss of enzyme activity even after long incubation times. Adsorption on ionic supports resulted in low-activity biocatalysts, and pH or temperature stability were not improved. Covalent attachment resulted in biocatalysts with decreased activity, but with a major impact on thermostability. The nature of the support was decisive on the catalytic activity of the enzyme by the substrate pNPP. Ionic and covalent supports improved more than 4-fold the enzyme–substrate affinity compared to free lipase, but for the hydrophobic support, no difference was noted. Due to high activity and possibility of stabilization of *M. spathulata* R25L270 lipase in the support Octyl-Sepharose, it was selected to evaluate the performance of the enzyme in the hydrolysis of sardine oil. The derivative showed selectivity for generation of EPA and DHA from sardine oil, and the selectivity could be improved by altering the pH of the reaction medium. The maximum selectivity (7.53) was found at pH 5.0. This study shows for the first time that the properties of *M. spathulata* R25L270 lipase can be modulated by directed immobilization.

## Figures and Tables

**Table 1:** Hydrolytic activity and immobilization parameters of *M. spathulata* R25L270 lipase on different supports.

Immobilization Method	Derivative	Hydrolytic Activity (U/g)	Immobilization Yield (%)	Recovered Activity (%)
Hydrophobic Adsorption	Butyl-Sepharose	5.82 ± 0.11	64.80	95.80
	Phenyl-Sepharose	5.33 ± 0.06	52.30	87.80
	Octyl-Sepharose	3.87 ± 0.02	63.98	63.80
	Octyl + polyethyleneimine (PEI)	4.41± 0.08	63.98	72.55
	Octyl + Trehalose	3.94 ± 0.05	63.98	64.92
Ionic Adsorption	MANAE (Monoaminoethyl-*N*-aminoethyl)-Agarose	1.32 ± 0.23	63.97	21.80
	DEAE-(Diethylaminoethyl)-Agarose	2.46 ± 0.38	75.20	40.51
	SP-(Sulfopropyl)-Sepharose	1.10 ± 0.04	22.29	18.19
Covalent Attachment	CNBr-(Cyanogenogen Bromide)-activated Sepharose	1.38 ± 0.02	36.27	22.73

Activities were measured by the pNPP assay, described in the Section 3.3. The activity of soluble lipase offered for immobilization was considered 100%. Parameters were calculated as described in Section 3.6.

**Table 2:** Kinetic constants of *M. spathulata* R25L270 lipase immobilized on different supports, determined by hydrolysis of pNPP.

Derivative	Michaelis Constant K_M_ (mM)	Maximum Velocity V_max_ (µmoles/min)	*R*^2^
Free	1.92	3.15	0.98
Octyl-Sepharose	1.35	2.48	0.99
Octyl + PEI	1.38	2.38	0.97
Octyl + Trehalose	1.66	2.79	0.99
DEAE-Agarose	0.40	2.01	0.98
CNBr-activated Sepharose	0.10	1.03	0.99

**Table 3:** pH stability of free and immobilized lipase at 25 °C. Values are the average of three independent replicates; error bars represent average ± one standard deviation.

	Residual Activity (%)		
Immobilized Derivative	pH 5.0	pH 7.0	pH 9.0
Free	62 ± 1	102 ± 2	105 ± 1
Butyl-Sepharose	0	96 ± 13	99 ± 14
Phenyl-Sepharose	85 ± 4	91 ± 11	106 ± 1
Octyl-Sepharose	100 ± 8	120 ± 4	113 ± 10
Octyl + PEI	96 ± 1	108 ± 5	112 ± 7
Octyl + Trehalose	89 ± 15	128 ± 18	105 ± 1
DEAE-Agarose	0	76 ± 1	104 ± 5
CNBr-activated Sepharose	0	122 ± 16	121 ± 15

**Table 4:** Kinetic parameters of free and immobilized *M. spathulata* R25L270 lipase thermal stability at 60 °C and pH 7.0.

Derivative	K_d_ (h^−1^)	*t*_1/2_ (h)	Stabilization Factor (SF)	*R*^2^
Free	1.72	0.40	-	0.99
Octyl-Sepharose	0.17	4.15	10.30	0.99
Butyl-Sepharose	0.65	1.06	2.63	0.98
Phenyl-Sepharose	0.29	2.39	5.92	0.98
DEAE-Agarose	2.21	0.31	0.77	1.00

**Table 5:** Parameters of fish oil hydrolysis with *M. spathulata* R25L270 lipase immobilized on ctyl-Sepharose.

	Selectivity ^a^		
Derivative	pH 5.0	pH 7.0	pH 9.0
Octyl-Sepharose	7.53	3.33	3.03
Octyl + PEI	6.05	4.14	2.39
Octyl + Trehalose	4.34	4.23	2.83

Reaction was carried out in an aqueous/organic biphasic system, with (50 mM McIlvaine buffer for pH 5.0; 50 mM sodium phosphate buffer for pH 7.0; and 50 mM bicarbonate buffer for pH 9.0)/cyclohexane, at 37 °C and 150 rpm. ^a^ Selectivity is expressed as the ratio between % of hydrolyzed EPA and % of hydrolyzed DHA. All parameters were calculated after 24 h of reaction.

## Supplementary Materials

Supplementary File 1

## Abbreviations

SF	Stabilization factors
EPA	eicosapentaenoic acid
PUFA	polyunsaturated fatty acids
DHA	docosahexaenoic acid
pNPP	*p*-nitrophenylpalmitate
pNP	*p*-nitrophenol
PEI	polyethyleneimine
CNBr	Cyanogen bromide-activated Sepharose
Y	Immobilization yield
RA	recovered activity
K_d_	deactivation rate constant
*t*_1/2_	half-life time
MANAE	monoaminoethyl-*N*-aminoethyl
DEAE	diethylaminoethyl
SP	sulfopropyl
