# Supplementary material for: Immobilization of Moniliella spathulata R25L270 Lipase on Ionic, Hydrophobic and Covalent Supports: Functional Properties and Hydrolysis of Sardine Oil
Source: Molecules. 2017 Sep 25;22(10):1508. doi: 10.3390/molecules22101508 (PMC6151709; doi:10.3390/molecules22101508)
Supplement: Supplementary File 1 [file molecules-22-01508-s001.zip › Supplementary file for calculating the kinetic parameters.pdf]

Supplementary file for calculating the kinetic parameters

Catalytic Properties

Values for apparent K_M and V_{max} were calculated based on Michaelis–Menten equation using the computational program Sigma Plot software. 10.0.

Table Suplemmentary 1: Kinetic constants of *M. spathulata* R25L270 lipase immobilized on different supports determined by hydrolysis of *p*-NPP. Values are the average of three independent replicates with coefficient of variation less than 10%

**Substrate**	**V (µmoles.min^{-1})**					
**pNPP (mM)**	**DEAE-Agarose**	**CNBr activated**	**Free lipase**	**Octyl-Sepharose**	**Octyl+ PEI**	**Octyl + Trehalose**
0.0	0.00	0.00	0.00	0.00	0.00	0.00
0.1	0.52	0.54	0.15	0.17	0.18	0.16
0.2	0.61	0.68	0.25	0.32	0.33	0.29
0.3	0.91	0.79	0.40	0.49	0.46	0.45
0.4	0.89	0.81	0.51	0.59	0.59	0.55
0.5	1.15	0.84	0.72	0.67	0.64	0.625
0.6	1.04	0.84	0.71	0.65	0.53	0.66
0.7	1.38	0.88	0.91	0.87	0.84	0.92
0.8	1.38	1.02	1.02	1.05	1.05	1.04
0.9			0.89	1.02	1.04	1.04
1.0				1.07	1.05	1.04
