# Supplementary material for: Immobilization of Moniliella spathulata R25L270 Lipase on Ionic, Hydrophobic and Covalent Supports: Functional Properties and Hydrolysis of Sardine Oil
Source: Molecules. 2017 Sep 25;22(10):1508. doi: 10.3390/molecules22101508 (PMC6151709; doi:10.3390/molecules22101508)
Supplement: Supplementary File 1 [file molecules-22-01508-s001.zip › CNBr activated.pdf]

Nonlinear Regression **segunda-feira, março 02, 2015, 19:59:56**

Data Source: Cinética de Michaelis in Octil, PEI e Treolase Cinética
Equation: Single Substrate; Michaelis-Menten in enzyme kinetics
v=Vmax*S/(Km+S)

R	Rsqr	Adj Rsqr	Standard Error of Estimate
0,9900	0,9801	0,9772	0,0449

	Coefficient	Std. Error	t	P
Vmax	1,0369	0,0457	22,6873	<0,0001
Km	0,1002	0,0204	4,9163	0,0017

Analysis of Variance:

	DF	SS	MS
Regression	2	5,2437	2,6219
Residual	7	0,0141	0,0020
Total	9	5,2578	0,5842

Corrected for the mean of the observations:

	DF	SS	MS	F	P
Regression	1	0,6943	0,6943	344,1766	<0,0001
Residual	7	0,0141	0,0020		
Total	8	0,7084	0,0886		

Statistical Tests:

Normality Test (Shapiro-Wilk) Passed (P = 0,1017)

W Statistic= 0,8624 Significance Level = <0,0001

Constant Variance Test Passed (P = <0,0001)

Fit Equation Description:
[Variables]
S = col(35)
V = col(36)
' Weighting Functions
reciprocal_V=if(V<=0; 0/0; 1/V)
reciprocal_Vsquare=if(V<=0; 0/0; 1/V^2)
[Parameters]
Vmax = max(V)*2 "Auto {{previous: 1,03685}}
Km = x50(S;V;0,1) "Auto {{previous: 0,100224}}
[Equation]
v=Vmax*S/(Km+S)
fit v to V
"fit v to V with weight reciprocal_V
"fit v to V with weight reciprocal_Vsquare
[Constraints]
Vmax>0
Km>0

[Options]
tolerance=0,00001
stepsize=1
iterations=200

Number of Iterations Performed = 7
