# Supplementary material for: Immobilization of Moniliella spathulata R25L270 Lipase on Ionic, Hydrophobic and Covalent Supports: Functional Properties and Hydrolysis of Sardine Oil
Source: Molecules. 2017 Sep 25;22(10):1508. doi: 10.3390/molecules22101508 (PMC6151709; doi:10.3390/molecules22101508)
Supplement: Supplementary File 1 [file molecules-22-01508-s001.zip › DEAE - Agarose.pdf]

Nonlinear Regression **terça-feira, março 03, 2015, 15:29:05**

Data Source: Cinética de Michaelis in Octil, PEI e Treolase Cinética
Equation: Hyperbola; Single Rectangular, 2 Parameter in Tempo de meia vida
f = a*x/(b+x)

**R**	**Rsqr**	**Adj Rsqr**	**Standard Error of Estimate**
0,9762	0,9530	0,9462	0,1031

	**Coefficient**	**Std. Error**	**t**	**P**
a	2,0097	0,3064	6,5592	0,0003
b	0,4028	0,1371	2,9369	0,0218

Analysis of Variance:

	**DF**	**SS**	**MS**
Regression	2	8,4426	4,2213
Residual	7	0,0744	0,0106
Total	9	8,5171	0,9463

Corrected for the mean of the observations:

	**DF**	**SS**	**MS**	**F**	**P**
Regression	1	1,5078	1,5078	141,8238	<0,0001
Residual	7	0,0744	0,0106		
Total	8	1,5823	0,1978		

Statistical Tests:

Normality Test (Shapiro-Wilk) Passed (P = 0,5464)

W Statistic= 0,9366 Significance Level = <0,0001

Constant Variance Test Passed (P = 0,6758)

Fit Equation Description:
[Variables]
x = col(1)
y = col(2)
reciprocal_y = 1/abs(y)
reciprocal_ysquare = 1/y^2
reciprocal_pred = 1/abs(f)
reciprocal_predsqr = 1/f^2
[Parameters]
a = max(y) "Auto {{previous: 2,0097}} {{MinRange: -114,522}} {{MaxRange: 343,566}}
b = if(x50(x;y;0,1)<>0; x50(x;y;0,1); 1) "Auto {{previous: 0,402753}} {{MinRange: -0,209038}} {{MaxRange: 0,627114}}
[Equation]
f = a*x/(b+x)
fit f to y
"fit f to y with weight reciprocal_pred
"fit f to y with weight reciprocal_predsqr
"fit f to y with weight reciprocal_y

"fit f to y with weight reciprocal_ysquare
[Constraints]
[Options]
tolerance=0,0000000001
stepsize=1
iterations=200

Number of Iterations Performed = 11
