# Supplementary material for: Immobilization of Moniliella spathulata R25L270 Lipase on Ionic, Hydrophobic and Covalent Supports: Functional Properties and Hydrolysis of Sardine Oil
Source: Molecules. 2017 Sep 25;22(10):1508. doi: 10.3390/molecules22101508 (PMC6151709; doi:10.3390/molecules22101508)
Supplement: Supplementary File 1 [file molecules-22-01508-s001.zip › Free Lipase.pdf]

Nonlinear Regression **segunda-feira, março 02, 2015, 20:37:03**

Data Source: Cinética de Michaelis in Octil, PEI e Treolase Cinética
Equation: Single Substrate; Michaelis-Menten in enzyme kinetics
v=Vmax*S/(Km+S)

R	Rsqr	Adj Rsqr	Standard Error of Estimate
0,9831	0,9665	0,9623	0,0678

	Coefficient	Std. Error	t	P
Vmax	3,1521	1,3134	2,4000	0,0432
Km	1,9183	1,0737	1,7865	0,1118

Analysis of Variance:

	DF	SS	MS
Regression	2	4,1634	2,0817
Residual	8	0,0368	0,0046
Total	10	4,2002	0,4200

Corrected for the mean of the observations:

	DF	SS	MS	F	P
Regression	1	1,0617	1,0617	230,9435	<0,0001
Residual	8	0,0368	0,0046		
Total	9	1,0985	0,1221		

Statistical Tests:

Normality Test (Shapiro-Wilk) Passed (P = 0,4131)

W Statistic= 0,9264 Significance Level = <0,0001

Constant Variance Test Passed (P = <0,0001)

Fit Equation Description:
[Variables]
S = col(16)
V = col(17)
' Weighting Functions
reciprocal_V=if(V<=0; 0/0; 1/V)
reciprocal_Vsquare=if(V<=0; 0/0; 1/V^2)
[Parameters]
Vmax = max(V)*2 ''Auto {{previous: 3,15215}}
Km = x50(S;V;0,1) ''Auto {{previous: 1,91828}}
[Equation]
v=Vmax*S/(Km+S)
fit v to V
''fit v to V with weight reciprocal_V
''fit v to V with weight reciprocal_Vsquare
[Constraints]
Vmax>0
Km>0

[Options]
tolerance=0,00001
stepsize=1
iterations=200

Number of Iterations Performed = 9
