# Supplementary material for: Immobilization of Moniliella spathulata R25L270 Lipase on Ionic, Hydrophobic and Covalent Supports: Functional Properties and Hydrolysis of Sardine Oil
Source: Molecules. 2017 Sep 25;22(10):1508. doi: 10.3390/molecules22101508 (PMC6151709; doi:10.3390/molecules22101508)
Supplement: Supplementary File 1 [file molecules-22-01508-s001.zip › Octyl + Trehalose.pdf]

Nonlinear Regression **segunda-feira, março 02, 2015, 20:32:15**

Data Source: Octil e Derivados CERTO in Octil, PEI e Treolase Cinética
Equation: Single Substrate; Michaelis-Menten in enzyme kinetics
v=Vmax*S/(Km+S)

R	Rsqr	Adj Rsqr	Standard Error of Estimate
0,9911	0,9822	0,9797	0,0481

	Coefficient	Std. Error	t	P
Vmax	2,7911	0,6766	4,1250	0,0044
Km	1,6618	0,5606	2,9645	0,0210

Analysis of Variance:

	DF	SS	MS
Regression	2	3,3391	1,6696
Residual	7	0,0162	0,0023
Total	9	3,3553	0,3728

Corrected for the mean of the observations:

	DF	SS	MS	F	P
Regression	1	0,8926	0,8926	386,5755	<0,0001
Residual	7	0,0162	0,0023		
Total	8	0,9088	0,1136		

Statistical Tests:

Normality Test (Shapiro-Wilk) Passed (P = 0,3529)

W Statistic= 0,9151 Significance Level = <0,0001

Constant Variance Test Passed (P = 0,0200)

Fit Equation Description:

```
[Variables]
S = col(10)
V = col(11)
' Weighting Functions
reciprocal_V=if(V<=0; 0/0; 1/V)
reciprocal_Vsquare=if(V<=0; 0/0; 1/V^2)
[Parameters]
Vmax = max(V)*2 ''Auto {{previous: 2,79107}}
Km = x50(S;V;0,1) ''Auto {{previous: 1,66179}}
[Equation]
v=Vmax*S/(Km+S)
fit v to V
''fit v to V with weight reciprocal_V
''fit v to V with weight reciprocal_Vsquare
[Constraints]
Vmax>0
Km>0
```

[Options]
tolerance=0,00001
stepsize=1
iterations=200

Number of Iterations Performed = 9
