# Supplementary material for: Immobilization of Moniliella spathulata R25L270 Lipase on Ionic, Hydrophobic and Covalent Supports: Functional Properties and Hydrolysis of Sardine Oil
Source: Molecules. 2017 Sep 25;22(10):1508. doi: 10.3390/molecules22101508 (PMC6151709; doi:10.3390/molecules22101508)
Supplement: Supplementary File 1 [file molecules-22-01508-s001.zip › Octyl+ PEI.pdf]

Nonlinear Regression **segunda-feira, março 02, 2015, 20:24:25**

Data Source: Data 2 in Octil, PEI e Treolase Cinética
Equation: Single Substrate; Michaelis-Menten in enzyme kinetics
v=Vmax*S/(Km+S)

**R**	**Rsqr**	**Adj Rsqr**	**Standard Error of Estimate**
0,9709	0,9427	0,9345	0,0823

	**Coefficient**	**Std. Error**	**t**	**P**
Vmax	2,3849	0,8893	2,6817	0,0315
Km	1,3820	0,7529	1,8356	0,1090

Analysis of Variance:

	**DF**	**SS**	**MS**
Regression	2	3,1525	1,5763
Residual	7	0,0474	0,0068
Total	9	3,1999	0,3555

Corrected for the mean of the observations:

	**DF**	**SS**	**MS**	**F**	**P**
Regression	1	0,7790	0,7790	115,1320	<0,0001
Residual	7	0,0474	0,0068		
Total	8	0,8264	0,1033		

Statistical Tests:

Normality Test (Shapiro-Wilk) Passed (P = 0,0002)

W Statistic= 0,6260 Significance Level = <0,0001

Constant Variance Test Passed (P = 0,0428)

Fit Equation Description:
[Variables]
S = col(6)
V = col(7)
' Weighting Functions
reciprocal_V=if(V<=0; 0/0; 1/V)
reciprocal_Vsquare=if(V<=0; 0/0; 1/V^2)
[Parameters]
Vmax = max(V)*2 "Auto {{previous: 2,38492}}
Km = x50(S;V;0,1) "Auto {{previous: 1,38197}}
[Equation]
v=Vmax*S/(Km+S)
fit v to V
"fit v to V with weight reciprocal_V
"fit v to V with weight reciprocal_Vsquare
[Constraints]
Vmax>0
Km>0

[Options]
tolerance=0,00001
stepsize=1
iterations=200

Number of Iterations Performed = 10
