# Supplementary material for: Immobilization of Moniliella spathulata R25L270 Lipase on Ionic, Hydrophobic and Covalent Supports: Functional Properties and Hydrolysis of Sardine Oil
Source: Molecules. 2017 Sep 25;22(10):1508. doi: 10.3390/molecules22101508 (PMC6151709; doi:10.3390/molecules22101508)
Supplement: Supplementary File 1 [file molecules-22-01508-s001.zip › Octyl-Sepharose.pdf]

Nonlinear Regression **segunda-feira, março 02, 2015, 20:22:23**

Data Source: Data 2 in Octil, PEI e Treolase Cinética
Equation: Single Substrate; Michaelis-Menten in enzyme kinetics
v=Vmax*S/(Km+S)

R	Rsqr	Adj Rsqr	Standard Error of Estimate
0,9913	0,9827	0,9802	0,0474

	Coefficient	Std. Error	t	P
Vmax	2,4794	0,4997	4,9621	0,0016
Km	1,3578	0,4017	3,3798	0,0118

Analysis of Variance:

	DF	SS	MS
Regression	2	3,4896	1,7448
Residual	7	0,0157	0,0022
Total	9	3,5053	0,3895

Corrected for the mean of the observations:

	DF	SS	MS	F	P
Regression	1	0,8920	0,8920	397,5430	<0,0001
Residual	7	0,0157	0,0022		
Total	8	0,9077	0,1135		

Statistical Tests:

Normality Test (Shapiro-Wilk) Passed (P = 0,0021)

W Statistic= 0,7140 Significance Level = <0,0001

Constant Variance Test Passed (P = 0,0583)

Fit Equation Description:

```
[Variables]
S = col(1)
V = col(2)
' Weighting Functions
reciprocal_V=if(V<=0; 0/0; 1/V)
reciprocal_Vsquare=if(V<=0; 0/0; 1/V^2)
[Parameters]
Vmax = max(V)*2 ''Auto {{previous: 2,47936}}
Km = x50(S;V;0,1) ''Auto {{previous: 1,35781}}
[Equation]
v=Vmax*S/(Km+S)
fit v to V
''fit v to V with weight reciprocal_V
''fit v to V with weight reciprocal_Vsquare
[Constraints]
Vmax>0
Km>0
```

[Options]
tolerance=0,00001
stepsize=1
iterations=200

Number of Iterations Performed = 9
